# Membrane-Assisted Methanol Synthesis Processes and the Required Permselectivity

**DOI:** 10.3390/membranes11080596

**Published:** 2021-08-06

**Authors:** Homa Hamedi, Torsten Brinkmann, Sergey Shishatskiy

**Affiliations:** Department of Process Engineering, Institute of Membrane Research, Helmholtz-Zentrum Hereon, Max-Planck-Straße P1, 21502 Geesthacht, Germany; homa.hamedimastanabad@hereon.de (H.H.); sergey.shishatskiy@hereon.de (S.S.)

**Keywords:** methanol synthesis, CO_2_ hydrogenation, membrane reactor, synthetic fuel, carbon capture, Aspen Custom Modeler, green fuel, carbon utilization

## Abstract

Water-selective membrane reactors are proposed in the literature to improve methanol yield for a standalone reactor. However, the methanol productivity is not a precise metric to show the system improvement since, with this approach, we do not consider the amount of energy loss through the undesirable co-permeation of H_2_, which could otherwise remain on the reaction side at high pressure. In other words, the effectiveness of this new technology should be evaluated at a process flowsheet level to assess its advantages and disadvantages on the overall system performance and, more importantly, to identify the minimum required properties of the membrane. Therefore, an equation-based model for a membrane reactor, developed in Aspen Custom Modeler, was incorporated within the process flowsheet of the methanol plant to develop an integrated process framework to conduct the investigation. We determined the upper limit of the power-saving at 32% by exploring the favorable conditions wherein a conceptual water selective membrane reactor proves more effective. Using these suboptimal conditions, we realized that the minimum required H_2_O/H_2_ selectivity is 190 and 970 based on the exergy analysis and overall power requirement, respectively. According to our results, the permselectivity of membranes synthesized for this application in the literature, showing improvements in the one-pass conversion, is well below the minimum requirement when the overall methanol synthesis process flowsheet comes into consideration.

## 1. Introduction

**Figure 1:**
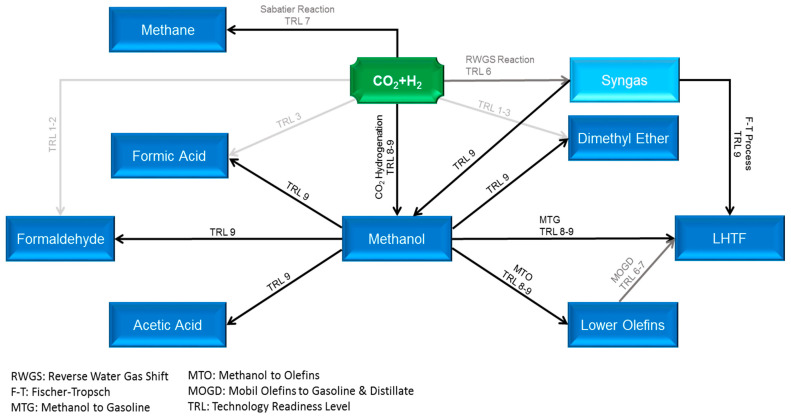
Carbon utilization pathways via thermocatalytic conversion with Technology Readiness Levels (TRLs) (blurred to sharp fonts denote low to high TRLs).

Carbon capture and utilization (CCU) is an economically and ecologically attractive solution to unravel the two contemporary global challenges, i.e., rapid climate change and future energy dilemma. However, the CCU cycle cannot be fully composed, unless the captured CO_2_ consolidates itself as major building blocks for the production of diverse high-demand CO_2_-based fuels, such as methane, methanol, and liquid hydrocarbon transportation fuels (LHTF), as well as chemicals. With almost 99 Mt global demand in 2020, methanol is identified as a pivotal intermediate for production of manifold chemicals (dimethyl ether, formaldehyde, formic acid, lower olefins, acetic acid, and higher alcohols), and LHTFs via the so-called methanol-to-gasoline (MTG) and Mobil olefins-to-gasoline-and-distillate (MOGD) technologies, instead of the traditional Fischer–Tropsch (FT) synthesis approach, as shown in Figure 1 [1,2,3,4]. Based on the premises, green methanol has substantial potential to shortly emerge as one of the key pieces of the carbon cycle puzzle; however, its production via CO_2_ hydrogenation is limited by equilibrium conversion under relevant process conditions and suffers from a high amount of water produced, which causes catalyst thermal deactivation. The in situ removal of water from the reaction system not only mitigates the two aforesaid problems but also intensifies the reaction rate and offers a unique opportunity to merge reaction and separation into one single piece of equipment with potential savings in capital and operating expenditures. This tactic can be carried out through the incorporation of membrane separation technology into the reaction system.

The first membrane reactor (MR) for methanol synthesis was proposed by Struis et al. [5]. The authors reported that the reactor equipped with a Nafion membrane outperforms conventional reactors (CR) with a 2.5% improvement in one-pass conversion. However, the membrane material was not sufficiently resistant to the temperatures at which the commonly used catalyst is active. A theoretical study of zeolite-based MRs with two hypothetical sets of species’ permeances was performed by Barbieri et al. [6]. The methanol yield increased from 5.8% to 13.7% in the best scenario of the MR system. A theoretical and experimental investigation was performed by Chen and Yuan [7]. They developed an isothermal MR for methanol synthesis utilizing a silicone rubber/ceramic composite membrane. They claimed a 22% enhancement in the reaction conversion. Gallucci et al. exploited zeolite membranes to experimentally study their impact on the isothermal reactor performance [8]. The results showed improvements in both selectivity and conversion. Raso et al. studied various zeolite membranes (zeolite A, mordenite, zeolite T, chabazite and Ti-chabazite) for the methanol production application [9]. Zeolite A was reported as the best choice for this application. The experimental results revealed that while this type of membrane increases the CO_2_ conversion, it decreases the methanol selectivity. A bifunctional catalytic MR based on a zeolite LTA was proposed by Yeu et al. [10]. Recently, a polymer–ceramic composite membrane was suggested by Juarez et al. as an alternative candidate for this application [11]. Due to the envisaged advantages of MR, it has been applied to several other CO_2_ conversion routes presented in Figure 1 [12,13,14,15]. 

Although several researchers have already investigated the feasibility of using MRs in the methanol synthesis application and unanimously concurred that MRs improve the system performance, the scope of their investigations was limited to laboratory-scale devices with an exclusive emphasis on the standalone reactor’s performance rather than the overall methanol process. While it was not clear if and to what extent these two metrics can represent the overall process performance enhancement, the one-pass reactor conversion and selectivity were used to show the improvement in the reaction system. More importantly, they completely overlooked the amount of the reactor feed pressure exergy (especially for hydrogen with high compression costs) sacrificed through the transmembrane pressure drop to promote the reactor conversion. This ignorance happens while the main advantage of MR is supposedly the reduction in the process energy demand through the recycle flowrate decrease. Finally, when the CO_2_ conversion rate increases but at the same time the methanol selectivity decreases, the use of the two metrics creates a predicament, whether the MR employment is advantageous or not [9,16]. 

In this work, we present a holistic framework by embedding a numerically based membrane reactor model, developed in Aspen Custom Modeler (ACM), within the entire methanol production process flowsheet simulated in Aspen Hysys V11. This integrated scheme enables us to accurately probe the role of an MR in the overall performance of an entire methanol process flowsheet, and analyze to what extent the employment of this technology impacts the overall utility demands of the plant. We determine in which heat transfer mode and process conditions the installation of an MR is appropriate. Finally, we suggest the minimum required H_2_O/H_2_ selectivity, above which an MR comes into effect and benefits the overall methanol process performance. The latter should be of high importance and interest to membrane material developers.

## 2. Process Description and Simulation

**Figure 2:**
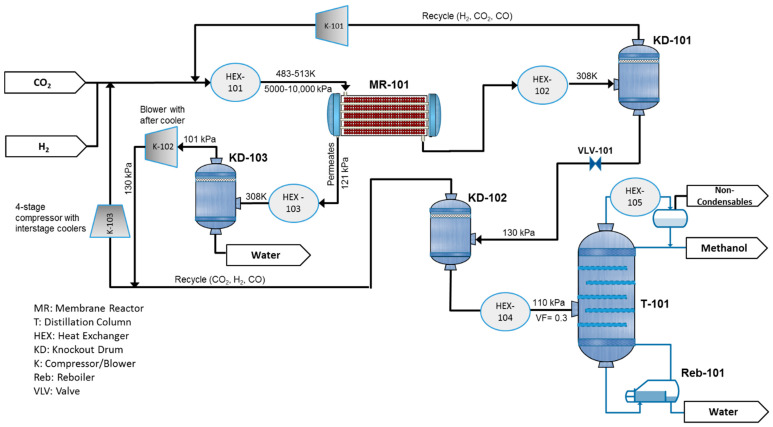
Process flow diagram for a methanol production plant aided with a water-selective membrane reactor (MR).

Figure 2 represents the process flow diagram of methanol production, which is aided by a water-selective MR. The plant is fed by pure CO_2_ and H_2_, which can be generated by water electrolysis using renewable energy sources. The feed is mixed with the recycle stream and heated in HEX-101 to the temperature of 483.15 K and 513.15 K for adiabatic and isothermal scenarios, respectively. Then, it is routed to the water-selective MR-101. The reactor product is cooled to 308.15 K to separate the unreacted gases from the condensable products, methanol and water, in the knockout drum (KD-101) and then recycled into the reactor after compression in K-101. The liquid product, crude methanol, is expanded through a valve (VLV-101) to 130 kPa pressure, and sent to the second separator (KD-102) to recover and further recycle the residual gases, which contain mostly CO_2_. The mixture of water and methanol is preheated and enters the distillation column T-101 to reach the product purification of 99% for both methanol and water. A very limited amount of CO_2_, along with other possible noncondensable impurities, is drawn from the column condenser as shown in the figure. The permeate coming from MR-101 is sent to HEX-101 and KD-103 to remove the produced water. The separated gases are compressed and recycled to the reactor. In the case of an infinitely water-selective membrane, K-102 is not necessary. Throughout this paper, a reactor with a membrane tube number equal to zero corresponds to a CR.

Membrane system separation is not suitable for sharp separation. Thus, to meet the product specification of 99% purity, the distillation tower is still required and industrially accepted.

Regardless of reactor designs, the overall productivity of a methanol production process is near 100% since the unreacted components and products can be almost thoroughly separated in the downstream equipment and recycled into the reactor. However, what is important here is how much energy/exergy is necessary for the overall conversion and separation.

**Figure A1:**
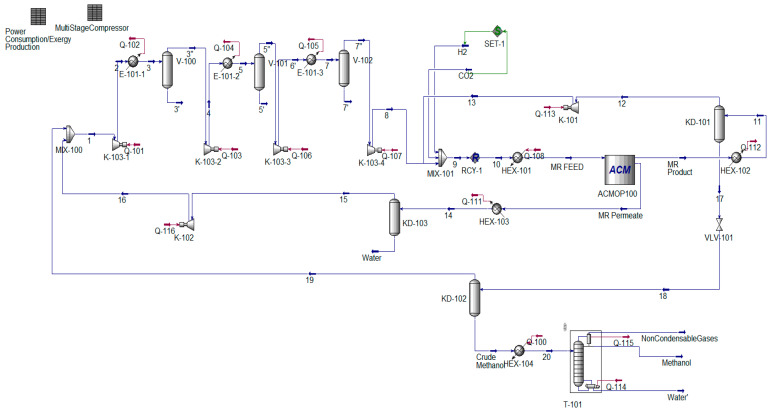
MR-based methanol production process flowsheet diagram simulated in Aspen HYSYS V11.

The process was simulated using Aspen HYSYS V11 (Aspen Technology Inc., Bedford, MA, USA). The Redlich–Kwong–Soave equation of state with modified Huron–Vidal mixing rules (RKSMHV2), developed in Aspen Plus, was imported and used as the fluid package [17]. The MR model, developed in ACM, is converted to an HYSYS package model and employed as an ACM operation unit in the process flowsheet (Appendix A, Figure A1). The details of the model are presented in our previous study with unrestricted access [16]. Though concentration polarization effects for gas permeation are reported negligible by several publications [18,19,20] due to the high diffusivity and low permeability of gases, we still need to clarify that the disregard of the phenomena in this primary assessment ensures that we are sufficiently cautious when presenting the lower limit for H_2_O/H_2_ selectivity. This is because, in the latter case, we overestimate the performance of the MR-based process during the comparison with the CR-based process. A crossflow pattern was assumed for the transmembrane mass transport. We used Ergun’s equation to calculate the pressure drop through the fixed bed reactor. For converging the recycles in the flowsheet shown in Appendix A. “Dominant Eigenvalue” should be used as the acceleration method for the single HYSYS recycle unit placed upstream of HEX-101. Due to the high nonlinearity of the problem, only the aforementioned method with at least 50 trials can solve the problem. The solution for the CR-based process should be set as an initial guess for the MR-based system. Additional specifications and process design data are provided in Section 5.

## 3. Reaction Kinetics

The methanol synthesis is a set of reversible reactions represented as follows [17]:

(1) $$CO\left(g\right)+2H_{2}\left(g\right)↔CH_{3}OH\left(l\right)\;\Delta H_{298K}=-128\;\frac{\mathrm{kJ}}{\mathrm{mol}}$$

(2) $$CO_{2}\left(g\right)+H_{2}\left(g\right)↔CO\left(g\right)+H_{2}O\left(g\right)\;\Delta H_{298K}=+41.19\;\frac{\mathrm{kJ}}{\mathrm{mol}}$$

(3) $$CO_{2}\left(g\right)+3H_{2}\left(g\right)↔CH_{3}OH\left(l\right)+H_{2}O\left(g\right)\;\Delta H_{298K}=-49.51\;\frac{\mathrm{kJ}}{\mathrm{mol}}\;$$

Van den Bussche and Froment’s kinetic model for a commercial Cu/ZnO/Al_2_O_3_ catalyst [21], which has been proven to describe the reaction system more accurately, was employed in our study [22]. Based on their model, the direct transformation of CO to methanol (Equation (1)) does not occur. We used the kinetic constants suggested by Mignard and Pritchard [23], which are valid for a pressure range of up to 7500 kPa [17]. Given the validity range for the parameters and the industrial operating pressure for methanol synthesis (5000–10,000 kPa) [17], the scope of this study was limited to 5000–7500 kPa. Moreover, though the use of MR at lower pressures can improve the reactor’s performance, it is still not satisfactory [16,22]. The reaction rates can be written as follows:

(4) $$r_{CH_{3}OH}=\frac{k_{1}p_{CO_{2}}p_{H_{2}}\;\left(1-\frac{1}{K_{eq1}}\;\frac{p_{H_{2}O}\;p_{CH_{3}OH}}{p_{H_{2}}^{3}\;p_{CO_{2}}}\right)}{{\left(1+k_{2}\frac{p_{H_{2}O}}{p_{H_{2}}}+k_{3}\;p_{H_{2}}^{0.5}+k_{4}\;p_{H_{2}O}\right)}^{3}}\;\rho_{cat}\;\left(1-\psi \right)\;[\frac{\mathrm{kmol}}{m^{3}\;h}]$$

(5) $$r_{RWGS}=\frac{k_{5}p_{CO_{2}}\;\left(1-K_{eq2}\;\frac{p_{H_{2}O}\;p_{CO}}{p_{H_{2}}\;p_{CO_{2}}}\right)}{\left(1+k_{2}\;\frac{p_{H_{2}O}}{p_{H_{2}}}+k_{3}\;p_{H_{2}}^{0.5}+k_{4}\;p_{H_{2}O}\right)}\;\rho_{cat}\;\left(1-\psi \right)\;\left[\frac{\mathrm{kmol}}{m^{3}\;h}\right]$$

(6) $$k_{i}=A_{i}\;exp\left(\frac{B_{i}}{RT}\right)$$

(7) $$log_{10}K_{eq1}=\frac{3066}{T}-14.592\;$$

(8) $$log_{10}K_{eq2}=\frac{2073}{T}-2.029\;$$

Symbols are described in Nomenclature and the required constants are presented in Table 1. We set the effectiveness factor at 1.0 due to the high pressure operating conditions and the small size catalysts of 2 mm, i.e., diffusion limitations are assumed negligible [24]. 

## 4. Membrane Properties

In the literature, various polymeric, ceramic, and zeolite membranes with hydrophilic structures have been suggested for MR-based methanol synthesis [5,6,7,8,9,25,26,27]. Due to the limited water permeances through these materials at elevated temperatures of the methanol reaction, the pressure on the permeate side should be set at the lowest possible, typically atmospheric pressure, to achieve the highest water permeation. At such high temperatures, for inorganic porous membranes, the Knudsen diffusion and molecular sieving are expected to be the dominating mechanisms for transmembrane permeation. In contrast, for membranes with a continuous polymeric selective layer, the gas permeances are determined by the solution–diffusion mechanism while the diffusion selectivity proves to be the dominator at elevated temperatures, especially in the case of glassy polymers. Among all the existing species in the reaction system, water and hydrogen are tightly competitive in permeation due to their small kinetic diameters, 0.265 and 0.289 nm, respectively. The co-permeation of hydrogen leads to very high penalties for the overall system since the recompression of this light gas from atmospheric pressure back to the reactor’s feed pressure is very costly. Thus, in general, high values of H_2_O/H_2_ permselectivity are demanded. However, the state-of-the-art polymeric membranes, which can be supported on porous ceramic or temperature stable, porous polymeric substrates, exhibit only 1–10 H_2_O/H_2_ permselectivity at the temperatures of interest [28,29,30]. On the other hand, ceramic membranes are also of poor permselectivity due to the degradation of the hydrophilic structure at high temperatures [28]. However, based on the experimental evaluation reported in the literature, zeolite membranes, in particular hydroxy sodalite (H-SOD) and mordenite, are proven to be the most promising materials for this particular application due to their higher H_2_O/H_2_ permselectivities and adequate thermal and mechanical stabilities under harsh conditions [27,28,31]. H-SOD with 0.27 nm openings is claimed to work solely based on molecular sieving and capable of selectively removing water vapor, with limited co-permeation of hydrogen depending on the reaction conditions [28,31]. Although this membrane has been employed in several theoretical studies for different reaction systems, its capability has never been tested and verified under the corresponding applied conditions [12], especially considering that the permeation properties of zeolite membranes depend on temperature, pressure, and the mixture composition [8]. However, a more target-based approach that can predict the minimum requirement of H_2_O/H_2_ permselectivity based on the methanol process simulation is expected to be of high importance and interest to membrane material developers for their future experiments. 

In this study, for the first part of our simulation, we assume an ideally water-selective MR, which is impermeable to H_2_, CO_2_, CO, and CH_3_OH, similar to what is claimed for H-SOD membranes [28,31]. The maximum H_2_O permeance of zeolite-based membrane in the literature, 10^−6^ mol/(s m^2^ Pa), was considered [28,32,33]. Based on these assumptions, we could demonstrate the highest energy saving potentials that can be achieved within a methanol synthesis process using MR technology. Moreover, this allows us to identify the circumstances in which MR is more effective. However, in the second part, the H_2_O/H_2_ permselectivity will be gradually reduced to find the minimum value that renders an MR-based process competitive with the CR-based counterpart. In both parts, the membrane is assumed to be completely impermeable to the other existing species (CO, CH_3_OH, and CO_2_). Later, we will provide a reason why this is a fair assumption.

## 5. Design Data and Specifications

The specifications that form the basis for the simulation are in Table 2. It is notable that the H_2_/CO_2_ feed ratio is marginally below the stoichiometric fraction since a very slight amount of the feed CO_2_, together with other possible noncondensable gases, is vented from the column overhead. Moreover, for a fair comparison between MR- and CR-based processes, the overall reactor volume (ORV), which is composed of the reactor’s and membrane’s volume, is kept constant, as in Equation (9) [33]. It is obvious that for a CR, the ORV and Reactor Volume are equal. This assumption is also valid for a retrofit scenario.

(9) ORV=Reactor Volume+Membrane Volume 

## 6. Results and Discussion

In this section, we evaluate and compare the performance of the methanol production processes, which include different reactor designs, namely CR and MRs with different membrane tube numbers (Nm). Two heat transfer scenarios are considered for the reactors. We used the two metrics of power consumption and exergy production as defined below.

(A) *Power Consumption*

(10) $$Power\;Consumption=\overset{103}{\underset{i=101}{\sum }}W_{K-i}$$

(B) *Exergy Production*

(11) $$Exergy\;Production=\;\overset{105}{\underset{i=101}{\sum }}Ex_{HEX-i}+\;Ex_{Reb-101}+\;Ex_{MR-101}-\overset{103}{\underset{i=101}{\sum }}W_{K-i}$$

(12) $$\mathrm{where}\;Ex_{MR-101}=\{\begin{array}{c} 0\;if\;adiabatic\\ \Delta H_{reactor}\left(1-\frac{T_{ambient}}{T_{reactor}}\right)if\;isothermal \end{array}$$

(13) $$\Delta H_{reactor}=H_{MR-101}^{in}-H_{MR-101}^{permeates}-H_{MR-101}^{product}$$

where W is compressor’s input power per CO_2_ feed mass flowrate (CFMF). Ex denotes the exergy production/consumption rate per CFMF for the respective equipment in Figure 2. Since the overall process always produces exergy, the summation is designated by Exergy Production. H is enthalpy rate per CFMF, and consists of both sensible enthalpy and the heat of formation as uniformly defined in the Aspen Engineering suite. 

It is notable that each metric presented in Equations (10) and (11) can alone serve as a basis for the comparison of CR- and MR-based processes. However, depending on the allowable tightness between the cold/hot composite curves during the heat integration practice, the subsequent exergy destruction can also be taken into account by giving a weighting factor to the first three terms in Equation (11) [34]. In this study, we used Equations (10) and (11) directly without any weighting factor. Furthermore, the aim of this study is not to compare the process performance under different feed pressures (or reactor’s operating pressure), but to assess the impact of the MR use on each process. Hence, for the comparison, we consider the metrics’ saving percentages that can be achieved by replacing the CR with different MRs, rather than their absolute values.

### 6.1. Adiabatic MR-Based Process 

**Figure 3:**
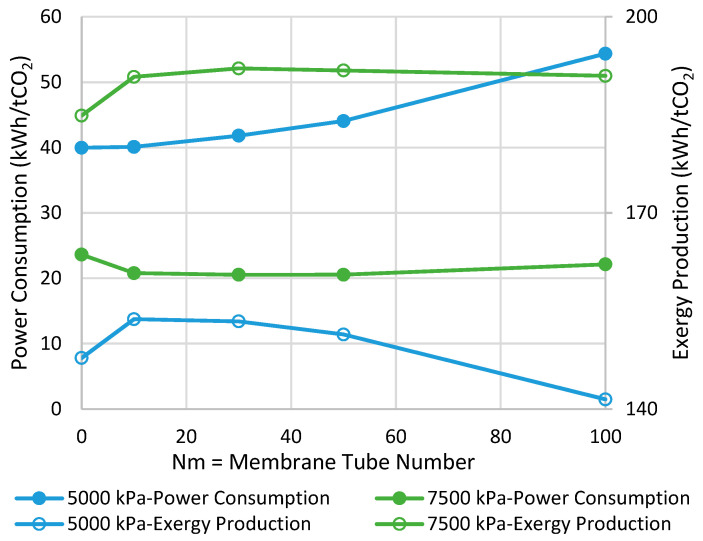
Comparison between adiabatic conventional reactors (CR) and MR-based processes with different membrane tube numbers using power consumption and exergy production (Nm = 0 corresponds to CR).

Figure 3 presents the comparison between MR- and CR-based processes in terms of power consumption and exergy production when the reactor operates with the adiabatic mode. As can be seen, at the pressure of 7500 kPa, the highest possible improvements are 13% and 4% in power consumption and exergy production, respectively, when Nm is 30. In contrast, at the pressure of 5000 kPa, no power consumption improvement is observed while a 4% enhancement in exergy production can be achieved at the best when Nm is 10. From Figure 3, we can conclude that at both operating pressures, increasing Nm is not always in a favor of the process and, thus, there is an optimal value for it. This tradeoff can be attributed to the following undesirable phenomena when the membrane area increases to possibly promote CO_2_-to-methanol conversion.

**Figure 4:**
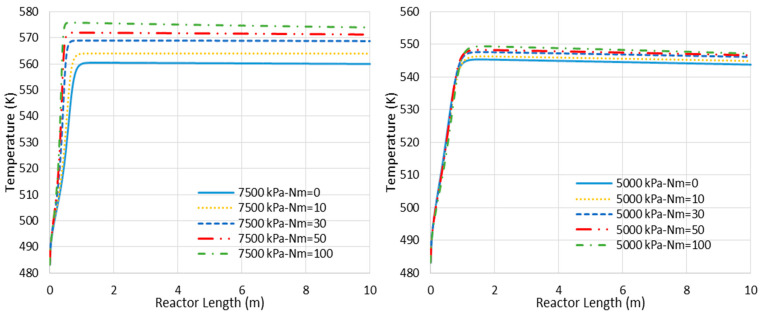
Temperature profile changes for CR- and MR-based processes with different membrane tube numbers (Nm) (Nm = 0 corresponds to CR).

**Figure 5:**
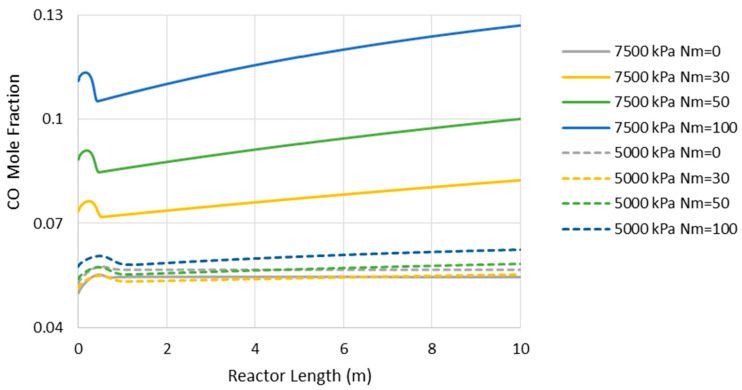
Carbon monoxide (CO) mole fraction through the adiabatic reactor’s length (Nm = 0 corresponds to CR).

(a) Higher loss of water from the reaction environment and the subsequent acceleration in temperature rise, as shown in Figure 4, which is not in a favor of the exothermic methanolation reaction.
(b) Reduction in CO_2_-to-methanol selectivity due to the aforementioned temperature rise and water removal [16] and, in turn, increase in CO concentration in the reactor environment, as presented in Figure 5. This increases the recompression power for the recycle.
(c) Higher exergy destruction of water through the isenthalpic transmembrane process.
(d) Losing reaction volume to accommodate more membrane tubes.
(e) A higher reactor pressure drop due to the membrane volumetric occupancy.

**Figure 6:**
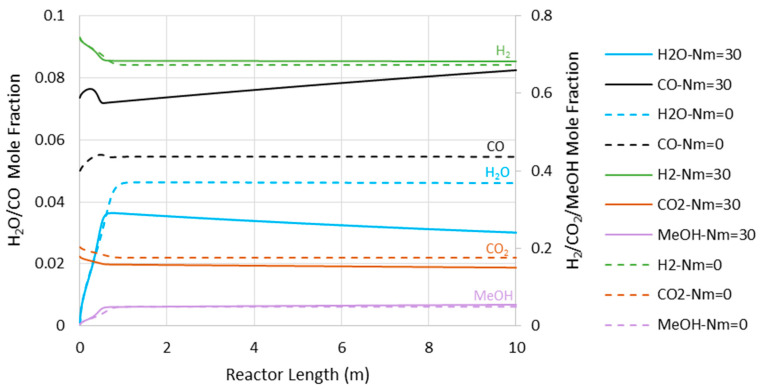
Species’ concentration profiles through the adiabatic reactor’s length at 7500 kPa (Nm = 0 corresponds to CR).

Figure 6 represents the concentration profiles for the existing species through the adiabatic CR and MR with Nm of 30 and 7500 kPa operating pressure (where the MR shows the highest impact). As shown, only the entrance section of the reactor is active. However, this active region of the reactor cannot be extended by the in situ water removal since the temperature rise as a dominating factor halts the reaction (see also Figure 4). After the temperature profile reaches 560 K for the CR and 569 K for the MR with Nm = 30, there are only slight changes in the species’ concentration, with some tendency toward CO production.

### 6.2. Isothermal MR-Based Process

Different heat transfer layouts, either proposed by the leading companies (Linde, Toyo, Methanol Casale, and Mitsubishi) or the literature, suggest various temperature profiles, from quasi-isothermal to adiabatic [22]. The optimal design of the heat transfer system takes a proper optimization investigation, which is out of the scope of the present work. However, in the previous section we realized that in an adiabatic MR the water removal amplifies the temperature rise, which curbs the positive impact of the membrane and inhibits the reaction. Consequently, if an MR avoids the adiabatic condition and approaches the isothermal operation, it should outperform the CR counterpart at highest. Therefore, we assume the isothermal reactor to ensure that we are on the safe side when proposing the minimal value for the lower limit of H_2_O/H_2_ selectivity.

**Figure 7:**
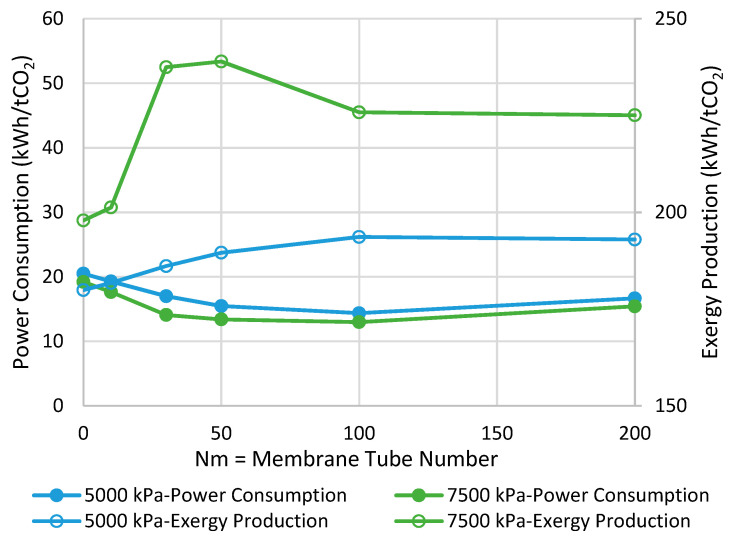
Comparison between isothermal CR- and MR-based processes with different membrane tube numbers using power consumption and exergy production (Nm = 0 corresponds to CR).

Figure 7 demonstrates the power consumption and exergy production for the methanol synthesis process using isothermal CR and MRs. The power consumption/exergy production can be improved by 30%/8% and 32%/21% for the operating pressure of 5000 and 7500 kPa, respectively, in their best-case scenario where the respective Nm is optimal. Comparing with the adiabatic scenarios, we can conclude that if the MR operates in the isothermal mode at 7500 kPa, the highest possible savings can be achieved in both of the metrics. 

**Figure 8:**
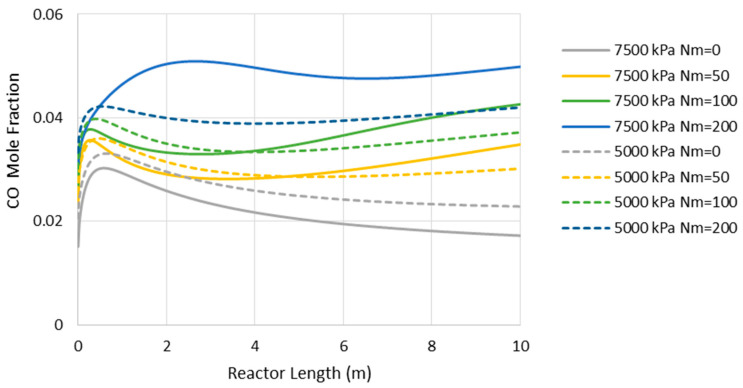
CO mole fraction through the isothermal reactor length (Nm = 0 corresponds to CR).

**Figure 9:**
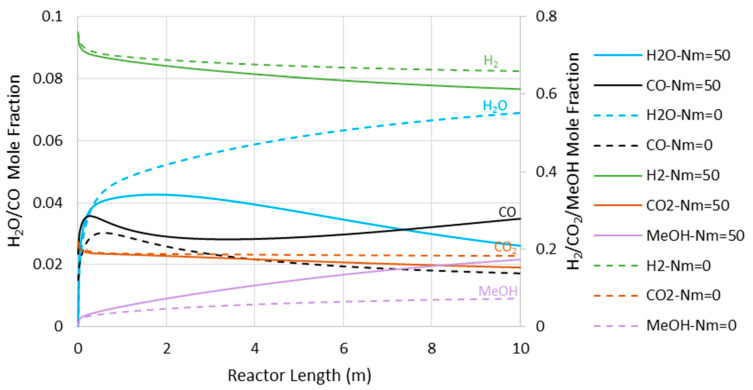
Species’ concentration profiles through the isothermal reactor length at 7500 kPa (Nm = 0 corresponds to CR).

Figure 8 presents the CO concentration in the reactor’s feed versus Nm at the pressures of 5000 and 7500 kPa. Similar to the adiabatic scenario (Figure 5), the CO feed mole fraction increases (in other words, methanol selectivity decreases) with Nm. However, compared to the adiabatic cases, CO exists at much lower levels, implying higher methanol selectivity. The latter can also be confirmed by comparing Figure 6 and Figure 9, where the CO concentrations are shown throughout the reactor’s length. Moreover, in contrast to the adiabatic scenario, for the isothermal condition, the reactor’s active region significantly extends to the almost full reactor length by the use of membranes. Nevertheless, despite the continuous water removal and isothermal condition, the driving force for the reaction decreases throughout the reactor’s length due to the increases in methanol concentrations. According to Equation (4), the reaction rate drops as the methanol partial pressure increases.

### 6.3. Minimum Requirement for H_2_O/H_2_ Membrane Permselectivity 

**Figure 10:**
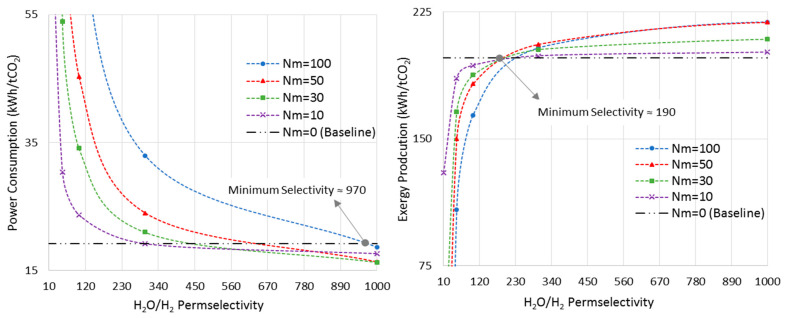
Power consumption/exergy production for isothermal-MR-based process at 7500 kPa with different Nm versus H_2_O/H_2_ permselectivity.

Figure 10 shows the power consumption and exergy production versus H_2_O/H_2_ permselectivity, in an isothermal MR-based process at the pressure of 7500 kPa, where the MRs showed the maximum improvements in both metrics (Section 6.2). As opposed to the last sections, in which the membrane was assumed to be permeable only to water, here we allow the H_2_O/H_2_ permselectivity to vary in order to realize the minimum requirement of this important parameter.

The CR-based process is considered the baseline. As earlier shown in Figure 7, at the pressure of 7500 kPa and the isothermal mode, the optimal Nm values are 100 and 50 for power consumption and exergy production, respectively. Thus, according to Figure 10, the minimum required H_2_O/H_2_ permselectivity is 970 and 190 when the power consumption and exergy production are taken into account, respectively. It is obvious that these are only the minimum values. However, in practice, since the MR-based process brings more complexity into the system, and the ideal isothermal condition may not be achieved in a large-scale reactor, this parameter should be much higher than its minimum in order for MR technology to be advantageous over the conventional design in terms of energy efficiency. Moreover, it is clear that the H_2_O/H_2_ permselectivity of polymeric and ceramic membranes, which is in the range of 1–10 [28], does not even satisfy the minimum requirement even though they enhance the standalone reactor’s conversion. To improve their selectivity, the latter calls for high-performance polymeric materials with a higher affinity toward water molecules at elevated temperatures or adoption of innovative techniques. Although zeolite membranes that have been utilized for methanol synthesis in the literature exhibit more promising characterizations for this application [9,27,28], their H_2_O/H_2_ permselectivities are still below the minimum. Moreover, H-SOD as the most promising zeolite-based membrane has been used for water separation at elevated temperatures only in theoretical works [12,28]. Hence, it still needs to be practically investigated under the methanol synthesis operating condition to study whether and to what extent H-SOD maintains its ideal molecular sieving feature.

According to Figure 10, as Nm decreases to lower values, there is less room for improvement. In other words, both power consumption and exergy production reach plateaus and show no improvements as the permselectivity increases.

Finally, considering the high values of the minimum required H_2_O/H_2_ permselectivity for this particular application, and the very small hydrogen kinetic diameter of 0.289 nm versus those of CO_2_, CH_3_OH, and CO with kinetic diameters of 3.3, 3.6, and 0.376 nm, respectively, the earlier assumption that the membrane is impermeable to the other existing species seems a fair conjecture.

## 7. Conclusions

MRs have established a proper niche in process intensification research for low conversion reaction system cases, including methanol synthesis. In this study, we compared MR-based and CR-based methanol production plants when the reactor pressure is in an industrial range of 5000–7500 kPa, using two key metrics of the “power consumption” and “exergy production”. We identified the conditions at which the use of MRs is more effective. The latter is the case when the MR operates in the isothermal condition and at higher pressures. For the best-case scenario of the adiabatic case, there were only 13% and 4% improvements in power consumption and exergy production, respectively, at the pressure of 7500 kPa. In contrast, for the isothermal mode, the power consumption/exergy production can be improved by 32% and 21 % at the same operating pressure, in the best-case scenario. Since an infinitely water-selective membrane with the permeance of 10^−6^ mol/(s m^2^ Pa) was assumed, these are the highest possible improvements in the process performance. We also showed that the minimum required H_2_O/H_2_ membrane permselectivity is 970 (190) by balancing the power consumption (exergy production) of the MR- and CR-based processes. This important information can shed light on future research directions. It is notable that since an MR brings more complexity into the system, these values are only considered as thresholds to make the MR-based process competitive with the CR-based counterpart. The current approach for the determination of the membrane’s minimum requirement is expected to be more broadly applied to the other membrane-reactor-aided processes proposed in the literature, such as syngas, methane, and Fischer–Tropsch syntheses.

## Figures and Tables

**Table 1:** Kinetic parameters for Equations (4)–(8) [16].

Parameters	Value
A_1_, kmol/(h kPa^2^ kg_cat_)	3.852 × 10^−4^
B_1_, kJ/kmol	40,000
A_2_	3453.38
B_2_, kJ/kmol	0
A_3_, kmol/(h kPa^0.5^ kg_cat_)	4.99 × 10^−2^
B_3_, kJ/kmol	17,197
A_4_, kmol/(h kPa kg_cat_)	6.62 × 10^−13^
B_4_, kJ/kmol	124,119
A_5_, kmol/(h kPa kg_cat_)	4.392 × 10^8^
B_5_, kJ/kmol	−98,084

**Table 2:** Design data and specifications.

Parameters	Value
CO_2_ feed flowrate, kmol/h	50
H_2_/CO_2_ feed ratio	2.974
H_2_/CO_2_ feed temperature, K	308
H_2_/CO_2_ feed pressure, kPa	5020 and 7520
reactor feed pressure, kPa	5000 and 7500
reactor’s inlet temperature for adiabatic reactors, K	483.15
reactor’s inlet temperature for nonadiabatic reactors, K	513.15
heat exchanger pressure drop, kPa	20
separator pressure drop, kPa	0
water product purity %	≥99.0
methanol product purity %	99.0
reactor length, m	10.0
reactor diameter, m	1.0
membrane water permeance, mol/(s m^2^ Pa)	10^−6^
membrane tube diameter, m	0.05
catalyst particle diameter, m	0.002
apparent catalyst density, kg_cat_/m^3^_cat_	1775
porosity	0.4
sphericity	1.0
number of distillation column trays	20
feed tray number for distillation column	10
ambient temperature, K	298.15

## Nomenclature

**Acronyms**	
CCU	carbon capture and utilization
CR	conventional reactor
CFMF	CO_2_ feed mass flowrate
FT	Fischer–Tropsch
H-SOD	hydroxy sodalite
LHTF	liquid hydrocarbon transportation fuels
MOGD	Mobil olefins to gasoline and distillate
MR	membrane reactor
MTG	methanol to gasoline
Nm	membrane tube numbers
ORV	overall reactor volume
RWGS	reverse water gas shift
tCO_2_	tonne (metric ton) of CO_2_
TRL	technology readiness level
**Variables/Parameters**	
Ex	exergy production/consumption rate per CO_2_ feed mass flowrate (kWh/tCO_2_)
H	enthalpy rate per CO_2_ feed mass flowrate (kWh/tCO_2_)
k	reaction rate’s parameters
$K_{\mathrm{eq}1}$	equilibrium constants for methanol reaction, Equation (7) (1/kPa^2^)
$K_{\mathrm{eq}2}$	equilibrium constants for reverse water gas shift reaction, Equation (8)
ORV	overall reactor volume (m^3^)
p	partial pressure (kPa)
r	reaction rate (kmol/m^3^ h)
R	universal gas constant (kJ/kmol K)
T	temperature (K)
$T_{\mathrm{ambient}}$	ambient temperature, 298.15 (K)
$T_{\mathrm{reactor}}$	isothermal reactor temperature (K)
W	compressor power per CO_2_ feed mass flowrate (kWh/tCO_2_)
$\rho_{\mathrm{cat}}$	catalyst’s density (kg/m^3^)
ψ	fixed bed porosity
$\Delta H_{\mathrm{reactor}}$	heat generation rate per CO_2_ feed mass flowrate for isothermal reactors (kWh/tCO_2_)
